# CXC chemokine receptor 4 expressed in T cells plays an important role in the development of collagen-induced arthritis

**DOI:** 10.1186/ar3158

**Published:** 2010-10-12

**Authors:** Soo-Hyun Chung, Keisuke Seki, Byung-Il Choi, Keiko B Kimura, Akihiko Ito, Noriyuki Fujikado, Shinobu Saijo, Yoichiro Iwakura

**Affiliations:** 1 Laboratory of Molecular Pathogenesis, Center for Experimental Medicine and Systems Biology, Institute of Medical Science, University of Tokyo, 4-6-1 Shirokanedai, Minato-ku, Tokyo 108-8639, Japan; 2 Current address: Department of Anatomy, College of Medicine, Korea University, 126-1, 5-Ga, Anam-Dong, Sungbuk-Gu, Seoul, Korea; 3 Division of Molecular Pathology, Institute of Medical Science, University of Tokyo, 4-6-1 Shirokanedai, Minato-ku, Tokyo 108-8639, Japan; 4 Core Research for Evolutional Science and Technology (CREST), Japan Science and Technology Agency, Saitama 332-0012, Japan

## Abstract

**Introduction:**

Chemokines and their receptors are potential therapeutic targets in rheumatoid arthritis (RA). Among these, several studies suggested the involvement of CXC chemokine 4 (CXCR4) and its ligand CXC ligand 12 (SDF-1) in RA pathogenesis. However, the role of these molecules in T-cell function is not known completely because of embryonic lethality of *Cxcr4- *and *Cxcl12-*deficient mice. In this report, we generated T cell-specific *Cxcr4*-deficient mice and showed that the CXCR4 in T cells is important for the development of collagen-induced arthritis (CIA).

**Methods:**

T cell-specific *Cxcr4*-deficient mice were generated by using the Cre-loxP system. Mice harboring loxP sites flanking exon 2 of the *Cxcr4*gene (*Cxcr4^flox/flox^*) were generated by homologous recombination and crossed with *Cre *transgenic mice expressing Cre recombinase under the control of *Lck *promoter (*Cxcr4^+/+^*/*Lck-Cre*mice) to generate T cell-specific *Cxcr4*-deficient mice (*Cxcr4^flox/flox^*/*Lck-Cre *mice). CIA was induced by immunization with chicken type II collagen and Complete Freund's Adjuvant (CFA).

**Results:**

The incidence, but not the severity, of CIA was significantly reduced in *Cxcr4^flox/flox^*/*Lck-Cre *mice compared with *Cxcr4^+/+^/Lck-Cre *mice. We found that the expression of CXCR4 was enhanced in activated T cells, and the migration of *Cxcr4*-deficient T cells toward SDF-1 was severely impaired. However, antibody production, cellular proliferative response, and cytokine production on treatment with type II collagen (IIC) were normal in these knockout mice, suggesting that CXCR4 is not involved in T-helper functions. Interestingly, the proportion of CXCR4-expressing T cells was much increased in affected joints compared with that in draining lymph nodes in CIA-induced mice, and distribution of *Cxcr4^flox/flox^*/*Lck-Cre *mouse-derived T cells into affected joints was suppressed compared with that in *Cxcr4^+/+^/Lck-Cre *T cells.

**Conclusions:**

These results indicate that CXCR4 expression in T cells is important for the development of CIA, by recruiting activated T cells toward inflammatory sites, and suggest that CXCR4 is a good target for the treatment of RA in humans.

## Introduction

Rheumatoid arthritis (RA) is an autoimmune disease affecting about 1% of the world population and characterized by chronic inflammation of multiple joints, proliferation of the synovial cells, and destruction of the cartilage and bone of the affected joints. Genetic factors and environmental agents are assumed to be involved in the development of disease, but the precise etiopathogenesis has not been elucidated completely [1,2].

To elucidate the complex RA pathogenesis, various disease models of RA have been developed [1,3]. Collagen-induced arthritis (CIA), one of the well-established animal models of RA, can be induced in mice by immunization with type II collagen (IIC) [4,5]. Although the concept that humoral and cellular immunity to IIC is crucial for the development of CIA is widely accepted, multiple chemokines and cytokines are also important for the pathogenesis.

CXC chemokine receptor (CXCR) 4 is a chemokine receptor expressed in various cells of the immune system and the central nervous system [6-8]. CXCR4 mediates migration of resting hematopoietic progenitors and B cells in response to its ligand, CXC ligand 12 (SDF-1), which is involved in various phenomena such as hematopoiesis and the development or survival of B cells [7,9]. Furthermore, the SDF-1-CXCR4 system is also suggested to be involved in the T-cell receptor (TCR) signaling [10] or cell migration or both [6].

Accumulating evidence suggests the involvement of the SDF-1-CXCR4 system in the pathogenesis of RA. CXCR4-expressing CD4^+^CD45RO^+ ^T cells are abundantly detected in the synovial tissues of RA patients [11]. T helper (Th) 1 cells, which are believed to be involved in the pathogenesis in part, are attracted by RA synovial fluid, and chemotaxis is interfered by anti-SDF-1 antibody *in vitro *[12]. SDF-1 is expressed at high levels in RA synovial tissues [13]. Furthermore, it is suggested that CXCR4 is important for T-cell retention in RA synovial tissues [11]. However, the importance of T cell-expressing CXCR4 in the development of RA and the functional role of CXCR4 in T cells still remain obscure, because many other cells, such as B cells and osteoclasts, also express CXCR4. Because null-knockout mice of *Cxcr4 *and *Cxcl12 *genes are embryonic lethal [14-16], it was difficult to elucidate the roles of this molecule in different cell types in the pathogenesis of diseases.

In this study, we generated T cell-specific *Cxcr4*-deficient mice and showed that the incidence of CIA was significantly decreased in these mice. Moreover, we confirmed that T cells migrate toward SDF-1 in a CXCR4-dependent manner *in vitro*, and CXCR4-expressing T cells were enriched in the affected joints during the development of CIA, suggesting involvement of T cell-expressing CXCR4 in the development of RA.

## Materials and methods

### Mice

**Figure 1:**
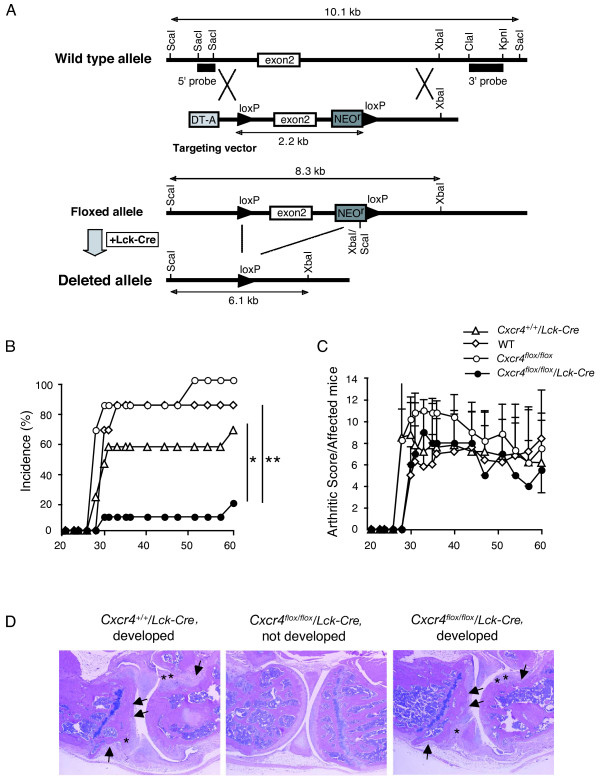
**The development of CIA is suppressed in *Cxcr4^flox/flox^*/*Lck-Cre *mice**. **(a) **Targeting strategy for the generation of conditional knockout mice of *Cxcr4*. T cell-specific *Cxcr4-*targeted (*Cxcr4^flox/flox^*/*Lck-Cre*) mice were generated as described in Materials and methods. **(b) **Incidence of CIA. **(c) **Severity of CIA. Mice were immunized with chicken IIC emulsified with CFA intradermally at triple sites into the back on day 0 and 21. Open circles, *Cxcr4^+/+ ^*mice (*n *= 6); open diamonds, *Cxcr4^flox/flox ^*mice (*n *= 6); open triangles, *Cxcr4^+/+^/Lck-Cre *mice (*n *= 9); solid circles, *Cxcr4^flox/flox^/Lck-Cre *mice (*n *= 11). Representative data obtained from two independent experiments are shown. Averages and SDs are indicated in c. **p < 0.01; *Cxcr4^flox/flox^/Lck-Cre *mice versus *Cxcr4^+/+ ^*and *Cxcr4^flox/flox ^*mice. *p < 0.05; *Cxcr4^flox/flox^/Lck-Cre *mice versus *Cxcr4^+/+^*/*Lck-Cre *mice, by χ^2^-test. **(d)**Histologic changes of the joints after induction of CIA. After the inspection of the arthritis development shown in b and c, hindlimbs were removed at 60 days after the first immunization, and paraffin sections of joints were stained with hematoxylin and eosin. The histology of the joint of a IIC-immunized and arthritis-developed *Cxcr4^+/+^*/*Lck-Cre *mouse (*Cxcr4^+/+^*/*Lck-Cre*, developed) shows severe erosion of the bone (arrows) and hyperplasia of synovial membrane (*), whereas no such abnormalities are seen in the joints of *Cxcr4^flox/flox^*/*Lck-Cre *mice that did not develop arthritis (*Cxcr4^flox/flox^*/*Lck-Cre*, not developed). A *Cxcr4^flox/flox^*/*Lck-Cre *mouse that developed arthritis (*Cxcr4^flox/flox^*/*Lck-Cre*, developed) shows severe bone erosion (arrows) and pannus-like structure (*), comparable to those of control mice. × 40. Typical histologies are shown in 22 representative sections from three mice for each group.

*Cxcr4^flox/flox ^*mice were generated as described previously in detail [17]. In brief, 4.7 kb of mouse genomic fragment containing the second exon of the *Cxcr4 *gene was floxed by two loxP sites containing neomycin-resistant (Neo^r^) gene, and the diphtheria toxin A (DT-A) gene was inserted to the upstream of the 5' arm for negative selection (Figure 1a). Targeted E14.1 ES cells were aggregated with C57BL/6J embryos, and the chimeric mice were crossed with C57BL/6J mice to obtain a germline-transmitted mouse. After intercrossing this mouse with proximal *Lck *promoter (*Lck*)-*Cre *transgenic mice (C57BL/6J background, kindly provided by Dr. Junji Takeda, Osaka University) [18], *Cxcr4^flox/+^*/*Lck-Cre *mice were backcrossed to the DBA/1J mice (Charles River Laboratories) for eight generations. Then they were intercrossed to obtain *Cxcr4^flox/flox^*/*Lck-Cre*, *Cxcr4^+/+^*/*Lck-Cre*, and *Cxcr4^flox/flox ^*mice on the DBA/1J background. Unless otherwise indicated, *Cxcr4^+/+^*/*Lck-Cre *mice on the DBA/1J background were used as controls. Age- and gender-matched mice were used for all experiments. Mice were housed under specific pathogen-free conditions in an environmentally controlled clean room at the Center for Experimental Medicine and Systems Biology, Institute of Medical Science, University of Tokyo. Experiments were performed according to the ethical guidelines for animal experimentation, which was approved by the Institutional Animal Care and Use Committee (reference number A17-5) and the safety guidelines for gene manipulation.

### Collagen-induced arthritis

CIA was induced as described previously [19]. In brief, mice were immunized with 100 μl of 2 mg/ml chicken IIC (Sigma-Aldrich, St. Louis, MO) emulsified in Complete Freund's Adjuvant (CFA; Difco) intradermally at three sites near the base of the tail on day 0. On day 21, the mice were given booster injection with the same amount of IIC/CFA intradermally near the former injection sites.

### Clinical and histologic assessment of arthritis

Development of arthritis by macroscopic evaluation was determined as described previously [19]. For histologic assessment of arthritis, limbs were fixed with 10% neutral formalin and were decalcified with 5% formic acid 60 days after the first immunization, and embedded in paraffin. Then 5-μm slices were prepared. Sections were stained with hematoxylin-eosin (H&E).

### Analysis on IIC-specific T-cell response

IIC-specific T-cell proliferative response was performed as previously described [20]. In brief, inguinal and infra-axillary lymph nodes (LNs) were harvested from mice 7 days after the primary immunization with chicken IIC with CFA. A single-cell suspension was prepared, and 3 × 10^5 ^cells/well in 96-well flat-bottom plates (Falcon) were cultured in the absence or presence of 50, 100, or 200 μg/ml of heat-denatured chicken IIC at 37°C for 72 hours, followed by incorporation of 25 μCi/ml [^3^H]-thymidine (Amersham, Chalfont St. Giles, Buckinghamshire, UK) for 6 hours. Then, cells were harvested with a Micro 96 cell harvester (Skatron, Lier, Norway), and radioactivity was measured with Micro Beta (Pharmacia Biotech, Piscataway, NJ). The concentrations of IL-17A and IFN-γ in the culture supernatant were determined by commercially available ELISA kits (Ready-Set-Go! Mouse IL-17A ELISA from eBioscience and Mouse IFN-γ ELISA kit from R&D systems).

### Measurement of collagen-specific Ig titers

Ig titers in serum were determined, as previously described [20]. In brief, 60 days after the first immunizations with IIC/CFA, serum was collected. Falcon 3912 Micro Test III Flexible Assay Plates (BD Biosciences, San Diego, CA) were coated with 0.1 ml/well of 10 μg/ml IIC in PBS at 4°C overnight. After washing with PBS, serially diluted serum samples were applied and incubated at room temperature for 1 hour. Then the wells were washed with PBS-0.05% Tween20, followed by the addition of alkaline phosphatase-conjugated goat anti-mouse IgG and IgM (Zymed, San Francisco, CA). Alkaline phosphatase activity was measured with Substrate Phosphatase SIGMA104 (Sigma-Aldrich) as the substrate. Results are expressed by the absorbance at 415 nm.

### *In vitro *chemotaxis assay

Migration of T cells was analyzed as described elsewhere [21]. In brief, T cells were purified from single-cell suspension of LNs by removing B cells with microbead-conjugated anti-mouse B220 antibody (Miltenyi Biotec) and an Auto-MACS (Miltenyi Biotec). T cells were suspended in RPMI 1640 with 1% FCS, and 3 × 10^6 ^cells were applied onto micropore filters (3.2 μm diameter, 3-μm pore polycarbonate transwell culture insert; ChemoTx, Gaithersburg, NeuroProbe). In the lower chamber, SDF-1a was supplemented in a concentration of 100 ng/ml and cultured for 2.5 hours in a 5% CO_2 _incubator at 37°C. Then migrated cells in the lower chamber were counted with a flow cytometer (FACS Calibur; Becton Dickinson, San Jose, CA) for 30 seconds with medium flow speeds. All points were determined in triplicate.

### *In vivo *migration assay

T-cell migration *in vivo *was analyzed by the transfer of radioisotope-labeled T cells as follows. Recipient mice were immunized twice with IIC 3 weeks and 1 week before the T-cell transfer. As the donors, *Cxcr4^+/+^*/*Lck-Cre *mice and *Cxcr4^flox/flox^*/*Lck-Cre *mice were immunized only once, 1 week before transplantation. Then LNs and spleens were harvested from the donor mice, and T cells were purified by using anti-Thy1.2 microbeads (Miltenyi Biotech) and Auto-MACS. T cells from each genotype were labeled with either ^51^Cr (PerkinElmer Life & Analytical Sciences) or ^111^In-oxin (Nihon Medi+physics) as previously described [22,24]. In brief, T cells were cultured with the indicated radioisotope at room temperature for 15 minutes, and then at 37°C for 15 minutes, and 1 hour in fresh medium. To compensate for potential radioisotope-specific artifacts, each cell population was split into two parts, and one half was labeled with ^51^Cr, and the other half with ^111^In, and we carried out the same experiments by using different combination of radioisotopes. Cell suspensions containing equal numbers of radioisotope-labeled *Cxcr4^+/+^*/*Lck-Cre *T cells and *Cxcr4^flox/flox^*/*Lck-Cre *T cells were prepared, and 0.2 ml of 2 × 10^7 ^cells/ml cell suspensions was injected to the recipient mice. Twenty hours after injection, LNs and digital joints were harvested, and the radioactivities were measured by using a γ-counter (Wallac, Turku, Finland). Radioactivities of ^51^Cr and ^111^In were measured separately, and cell accumulation was estimated by the radioisotope counts. Cell migration was not affected by the radioisotopes used for the labeling.

### Flow cytometry

Single-cell suspensions were prepared from LNs, spleens, thymus glands, and joints, and stained with FITC-, PE-, PerCP-, APC-, or biotin-conjugated antibodies. Biotinylated antibodies were visualized with PE- or APC-conjugated streptavidin (BD Biosciences). To prepare single-cell suspensions from joints, joints were collected and digested with 200 units/ml of collagenase (type VIII, Sigma) and hyaluronidase (1.5 ng/ml) in FCS-supplemented RPMI1640 medium for 1.5 hour at 37°C with gentle mixing. Then cells were filtered with cell strainers (Falcon) and stained. Stained cells were analyzed with FACSCalibur and FlowJo software. Antibodies to CXCR4 (BD Pharmingen), CD44 (eBioscience), CCR6 (R&D Systems), CD62L (BD Pharmingen), CD4 (BD Pharmingen), and CD8 (BD Pharmingen) were used.

### Immunohistochemistry

For immunohistochemical analysis, hindlimbs from normal mice and CIA-affected mice were embedded in CMC gel with hexane, and cryostat sections (5 μm) were generated. The sections were fixed in cold acetone for 5 minutes and blocked with 2% bovine serum albumin (Sigma) in PBS with 0.05% Tween 20. Antibodies used were as follows; biotinylated rat anti-murine CXCR4 (BD Pharmingen), rabbit anti-murine CD3 (Abcam, Ab16044), biotinylated donkey anti-rat IgG (Jackson) to amplify the signal from CXCR4, streptavidin FITC (BD Pharmingen), and Cy3 goat anti-rabbit IgG (Jackson). Nuclei were stained with Hoechst 33258 (WAKO). The slides were visualized on a Nikon A1Rsi-TiE confocal microscope operated by NIS-Elements software (see also Additional file 1).

### Statistics

Student's *t*-test and the χ^2^-test were used for the statistical evaluation.

## Results

### T cell-specific *Cxcr4*-deficient mice were generated by crossing *Lck-Cre *transgenic mice with *Cxcr4^flox/flox ^*mice

To investigate the roles of CXCR4 in T cells, we generated T cell-specific conditional gene-targeted mice, as described in Materials and methods. Mice harboring lox P sites flanking the second exon of *Cxcr4 *gene (*Cxcr4^flox/flox ^*mice) were generated by homologous recombination (Figure 1a) and crossed with a *Lck-Cre *transgenic mouse [18]. Deletion of the *Cxcr4 *gene and CXCR4 protein in the thymus of *Cxcr4^flox/flox^/Lck-Cre *mice was confirmed (Additional file 2a, b). KO mice were born in the mendelian ratio, and no gross phenotypic abnormalities were observed. *Cre *recombinase was activated from the DN3 stage of thymic T-cell development in *Cxcr4^flox/flox^/Lck-Cre *mice (Additional file 2c). No significant abnormalities in T-cell number and population in LNs and spleen were found in *Cxcr4^flox/flox^*/*Lck-Cre *mice, although thymocytes were significantly reduced (Additional file 2d), indicating that CXCR4 deficiency does not affect peripheral T-cell development.

### The development of CIA is significantly impaired in *Cxcr4^flox/flox^*/*Lck-Cre *mice

We examined the effect of T cell-specific *Cxcr4 *deficiency in the development of CIA. The incidence of the CIA was significantly more decreased in *Cxcr4^flox/flox^*/*Lck-Cre *mice than in *Cxcr4^+/+ ^*mice, *Cxcr4^flox/flox ^*mice, or *Cxcr4^+/+^*/*Lck-Cre *mice (Figure 1b). Conversely, no significant difference was found in the severity score of the arthritic mice between *Cxcr4^flox/flox^*/*Lck-Cre *mice and *Cxcr4^+/+^*/*Lck-Cre *mice (Figure 1c). In the histologic analysis, affected joints of both control mice and *Cxcr4^flox/flox^*/*Lck-Cre*mice showed typical features of arthritis, characterized by marked perivascular infiltration of inflammatory cells, synovial hyperplasia, and bone erosion (Figure 1d). In the joints of nonarthritic *Cxcr4^flox/flox^*/*Lck-Cre *mice, these arthritic pathologies were not detected. These observations suggest that CXCR4 in T cells plays an important role in the development of arthritis.

### Thymocytes and LN T cells migrate toward SDF-1 in a CXCR4-dependent manner

**Figure 2:**
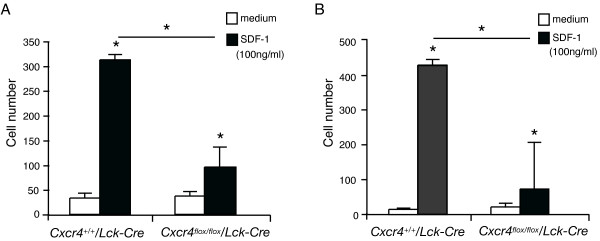
***Cxcr4^flox/flox^*/*Lck-Cre*mouse-derived T cells show impaired chemotaxis toward SDF-1**. The chemotaxis toward SDF-1 of T cells from *Cxcr4^+/+^*/*Lck-Cre *mice or *Cxcr4^flox/flox^*/*Lck-Cre *were assessed with *in vitro *chemotaxis assays, as described in Materials and methods. Representative results in at least two independent experiments are shown. **(a) **Chemotaxis of thymocytes to SDF-1 or medium was compared between *Cxcr4^+/+^*/*Lck-Cre *mice and *Cxcr4^flox/flox^*/*Lck-Cre *mice. *P < 0.05, by Student's *t*-test. **(b) **Chemotaxis of purified T cells from LNs was assessed. Results are shown as mean ± SD of triplicate determinations and are representative of at least two similar experiments. *P < 0.05, by Student's *t-*test Open columns indicate numbers of cells migrated into the chambers filled with medium only, and black columns, into the chambers with the medium supplemented with SDF-1 (100 ng/ml).

To elucidate functional roles of CXCR4 in T cells, we examined T-cell chemotaxis to SDF-1 by using chemotaxis chambers. Thymocytes from *Cxcr4^+/+^*/*Lck-Cre *mice efficiently migrated to SDF-1 (Figure 2a). In contrast, migration of *Cxcr4^flox/flox^*/*Lck-Cre *mouse-derived thymocytes was greatly reduced. Likewise, T cells purified from LNs of wild-type mice efficiently migrated toward SDF-1, whereas cells from *Cxcr4^flox/flox^*/*Lck-Cre *mice migrated less efficiently (Figure 2b), indicating that SDF-1-CXCR4-dependent chemotaxis is functional in T cells, similarly to hematopoietic cells and mature B cells [25,26].

### Humoral and cellular responses to IIC are normal in *Cxcr4^flox/flox^*/*Lck-Cre *mice

**Figure 3:**
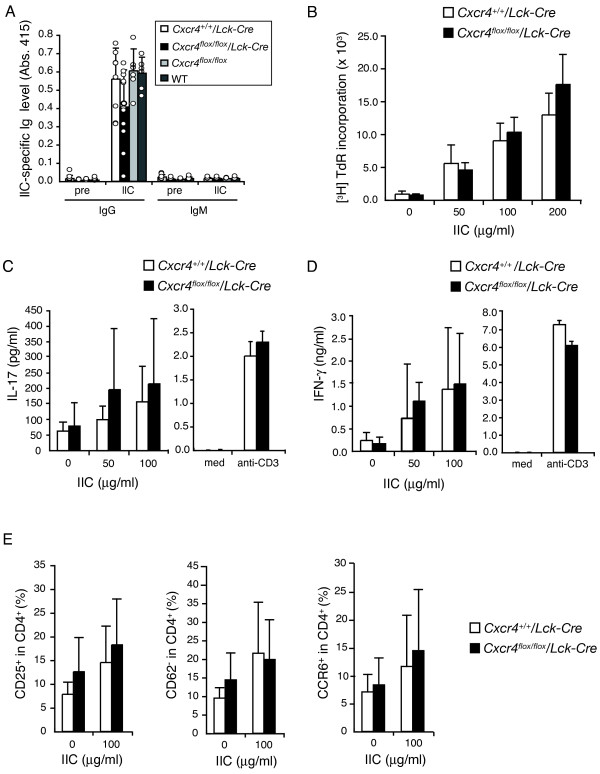
**Humoral and cellular immune responses against IIC are normal in *Cxcr4^flox/flox^*/*Lck-Cre *mice**. **(a) **IIC-specific IgG and IgM levels in the serum were determined with ELISA 60 days after immunization with IIC/CFA. Each circle represents an individual mouse, and averages and SDs are shown. *Cxcr4^+/+^*/*Lck-Cre *mice: open columns (*n *= 9); *Cxcr4^flox/flox^*/*Lck-Cre *mice: closed columns (*n *= 11); *Cxcr4^flox/flox ^*mice: light gray columns (*n *= 6); and WT DBA/1J mice: dark gray columns (*n *= 6). Data are representative of two independent experiments. **(b) **T-cell recall proliferative response toward IIC was assessed 1 week after immunization with IIC/CFA. LN cells from *Cxcr4^+/+^*/*Lck-Cre *or *Cxcr4^flox/flox^*/*Lck-Cre *mice were harvested and cultured in the presence or absence of 50, and 100, 200 μg/ml of denatured IIC at 37°C for 72 hours, and T-cell proliferation was assessed with [^3^H]-thymidine incorporation by using three wells for each mouse. The average and SD of four mice are shown for each column. Representative data from three independent experiments are shown. IL-17 **(c) **and IFN-γ **(d) **levels in the culture supernatants were measured with ELISA. Triplicate (proliferation, IFN-γ) or duplicate (IL-17) wells were used for each mouse, and averages and SDs of three mice are shown. Data are representative of at least two independent experiments. In the right columns, IL-17 (C) and IFN-γ (D) levels in cultures of LN cells from two immunized mice after stimulation with 1 μg/ml of anti-CD3 antibody at 37°C for 72 hours are shown. Averages and SDs of triplicate wells are shown, and data are representative of two independent experiments. **(e) **LN cells from immunized mice were pooled and stimulated with or without 100 μg/ml of IIC for 72 hours. The proportion of CD25^+^, CD62L^-^, and CCR6^+ ^cells in CD4^+ ^cells was analyzed with FACS. Data from three independent experiments were combined, and averages and SDs are shown.

As antibody production and cellular response to IIC are important for the development of CIA [5], we next analyzed the antibody titer in sera and the proliferative response of LN cells to IIC. Although IIC-specific IgG in the serum of *Cxcr4^flox/flox^/Lck-Cre *mice tended to decrease at day 60 of immunization, no significant differences between the control mice were found at days 7 and 60 (Figure 3a and Additional file 3a). In addition, no significant difference of LN cells was found in the proliferative response to anti-IgM antibodies (Additional file 3b). These data indicate that B-cell function is normal in *Cxcr4^flox/flox^*/*Lck-Cre *mice. To examine the cellular response to IIC, *Cxcr4^flox/flox^/Lck-Cre *mice and *Cxcr4^+/+^*/*Lck-Cre *mice were immunized with IIC with CFA, and LN cells from these mice were restimulated with IIC *in vitro*. The proliferative response to IIC was normal in *Cxcr4^flox/flox^/Lck-Cre *mouse-derived LN cells (Figure 3b), and levels of IL-17 and IFN-γ in the culture supernatant were also normal (left columns in Figure 3c and 3d). To confirm these results, we stimulated LN cells from immunized mice with anti-CD3 antibody instead of IIC in the same experimental settings and detected normal levels of IL-17 and IFN-γ in the culture supernatant (right columns in Figure 3c and 3d) in *Cxcr4^flox/flox^/Lck-Cre *mouse-derived LN cells. In addition, the expression levels of CD62L, a naïve T-cell marker, and CD25, a T cell-activation marker, and CCR6 for Th17 marker, were similar between *Cxcr4^flox/flox^/Lck-Cre *mice and *Cxcr4^+/+^/Lck-Cre *mice (Figure 3e and Additional file 4). As it was reported that pretreatment of T cells with SDF-1 enhances TCR-induced proliferative response [27,28], we examined the effect of SDF-1 treatment on the IIC-induced T-cell proliferation. However, no significant enhancement in T-cell proliferative response was observed in our experimental conditions (Additional file 5).

### CXCR4-expressing T cells are increased in the draining LNs of arthritic mice

**Figure 4:**
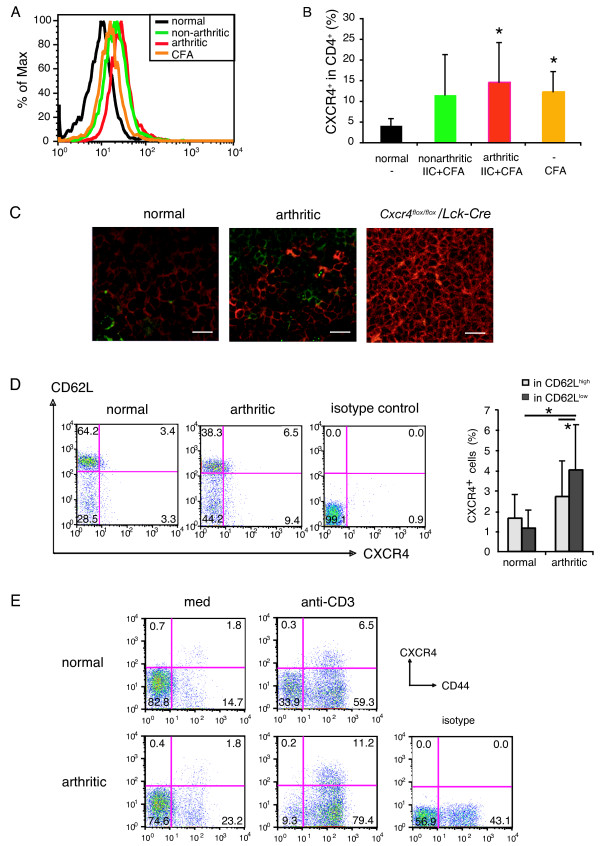
**CXCR4-expressing T cells are increased in LNs during the development of CIA**. DBA/1J mice were immunized with IIC and CFA, or CFA only, on day 0 and day 21. At day 30, arthritis development was determined, and LN cells were collected. **(a) **CXCR4 expression was analyzed with flow cytometry in CD4^+ ^T cells from IIC and CFA-immunized mice (arthritic and nonarthritic), CFA-immunized mice (nonarthritic), and normal nonimmunized DBA/1J mice. Representative data from two similar experiments are presented. **(b) **Proportion of CXCR4^+ ^cells in CD4^+ ^cells from normal DBA/1J mice without immunization (*n *= 3), IIC and CFA-immunized nonarthritic (*n *= 4) and arthritic (*n *= 7) and CFA-immunized nonarthritic (*n *= 7) mice are shown. Data from two similar experiments were combined. *P < 0.05 by Student's *t *test. **(c) **The expression of CXCR4 was analyzed with immunohistochemistry. CXCR4 was labeled with green fluorescence, and CD3 was labeled with red fluorescence. An increased number of CXCR4-expressing T cells is found in LNs from arthritic mice than those from normal mice. Representative figures among three sections from normal mice and three sections from arthritic mice are presented. White bar represents 20 μm. **(d) **CXCR4 and CD62L coexpression was analyzed in CD4^+ ^cells. Representative plots gated on CD4^+ ^cells are presented, and all the data from 10 normal nonimmunized mice and 11 IIC/CFA-immunized arthritic mice are summarized on the right. Averages and SDs are shown. *P < 0.05 by Student's *t *test. **(e) **LN cells from two nonimmunized normal or IIC/CFA-immunized arthritic mice were pooled and cultured in the absence or presence of 1 μg/ml of anti-CD3 antibody for 72 hours, and CXCR4 and CD44 expression was analyzed in CD4^+ ^cells. Representative plots gated on CD4^+ ^cells from two independent experiments are shown.

**Figure 5:**
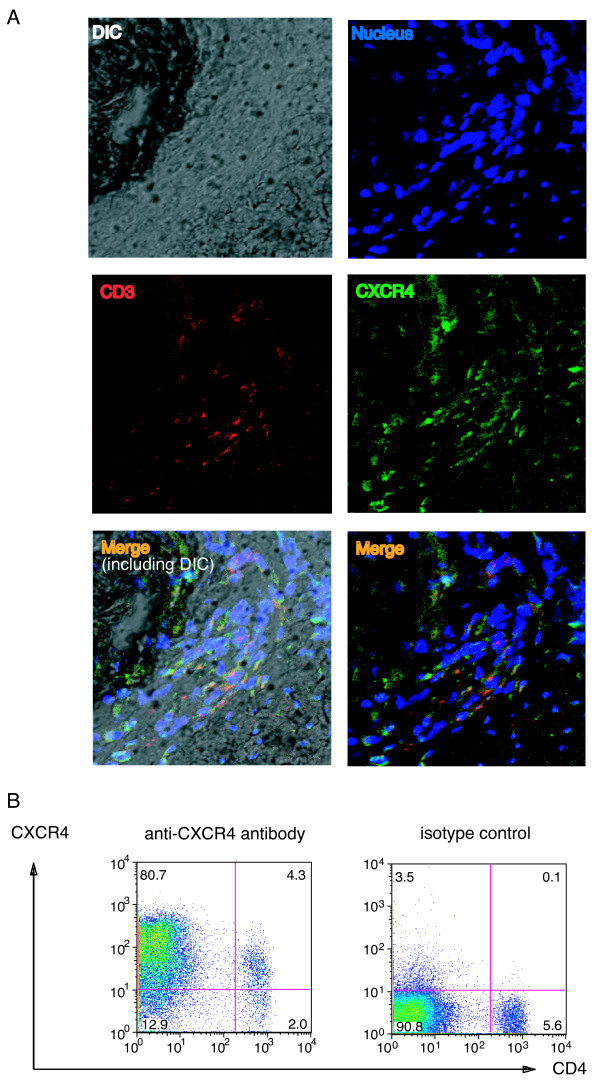
**CXCR4 is expressed in most of synovial T cells from arthritic mice**. Mice were induced CIA and CXCR4 expression in the affected joints was analyzed by immunohistochemistry. Cryostat sections of the limb joints from arthritic mice or normal mice were generated and stained with anti-CD3 and anti-CXCR4 antibodies. **(a) **Representative data among sections from three normal mice and three arthritic mice are shown. Data are summarized in Figure 6c. **(b) **CXCR4 expression on T cells was analyzed with FACS. As described in Materials and methods, affected limbs from CIA-developed mice were treated with hyaluronidase and collagenase to obtain single-cell suspensions, and stained with anti-CD4 and anti-CXCR4 antibodies. Representative data among three similar experiments are shown.

**Figure 6:**
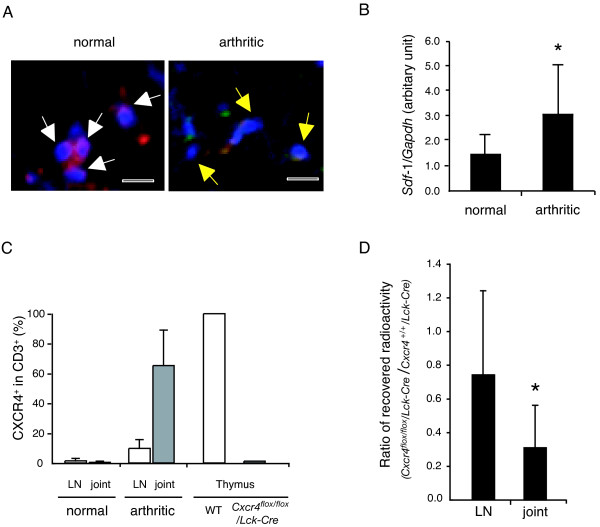
**T-cell accumulation in arthritic joints is dependent on CXCR4 expression in T cells**. **(a) **CXCR4 was expressed on CD3^+ ^T cells in arthritic joints. Cryostat sections from limbs of normal mice or arthritic mice were analyzed with immunohistochemistry. Red, CD3; green, CXCR4; and blue, nucleus. CD3^+^CXCR4^- ^cells are indicated with white arrows, and CD3^+^CXCR4^+ ^cells with yellow ones. White bars indicate 10 μm. Representative data from sections of three normal and three arthritic mice are shown. **(b) **The expression level of SDF-1 in joints was analyzed with real-time PCR. Normal, joints from nontreated DBA/1J mice (*n *= 10); arthritic, affected joints from CIA-induced mice (*n *= 12). Averages and SDs are shown. **(c) **CXCR4 expression in CD3^+ ^cells was summarized from the immunohistochemical analyses of three normal and three arthritic mice (shown in Figures 4c, 5a, and 6a), and the averages and SDs are presented. Sections of the thymus from a DBA/1J mouse and a *Cxcr4^flox/flox^*/*Lck-Cre *mouse were analyzed as the controls (indicated as Thymus WT or *Cxcr4^flox/flox^*/*Lck-Cre*). **(d) ***In vivo *migration of T cells into inflammatory sites during development of CIA was dependent on CXCR4 expression. T cells from IIC/CFA-immunized *Cxcr4^+/+^/Lck-Cre *mice (*n *= 4) and *Cxcr4^flox/flox^*/*Lck-Cre *mice (*n *= 4) were purified and differentially labeled with ^111^In and ^51^Cr, as described in Materials and methods. Cell suspension containing equal numbers of cells from each genotype was injected into CIA-induced recipient mice. After 20 hours, the radioactivities in limbs or LNs were measured with a gamma counter. From the percentages of radioactivities distributed in LNs or joints, the ratio of *Cxcr4^flox/flox^/Lck-Cre *mouse-derived T cells versus *Cxcr4^+/+^/Lck-Cre *mouse-derived T cells was calculated. Data from two similar experiments were combined, and averages and SDs of five recipient mice are presented.

Because CXCR4 was not involved in the humoral and cellular responses to IIC, we hypothesized that the CXCR4-mediated chemotaxis of T cells is involved in the development of CIA. To verify this possibility, we investigated the proportion of the CXCR4-expressing T cells in the LNs and the joints (Figure 4a-c, Figure 5, and Figure 6a-c). DBA/1J mice were immunized with IIC/CFA to induce arthritis, and the expression of CXCR4 was determined with FACS analysis. In cells from the draining LNs, a peak-shift of the intensity was observed in arthritic mice compared with nonarthritic mice, indicating that CXCR4 was induced in arthritic mice (Figure 4a, b, and Additional file 6). Furthermore, cryostat sections of LNs stained with anti-CD3 and anti-CXCR4 antibodies revealed that the number of CXCR4-expressing T cells was increased in the arthritic mice compared with nonarthritic mice (Figure 4c, summarized in Figure 6c).

The expression of CXCR4 also tended to increase in nonarthritic mice after IIC/CFA immunization, although the increase was not statistically significant (Figure 4a, b). The expression of CXCR4 increased in mice immunized with CFA only, but LN cells from these mice did not respond to stimulation by IIC or develop arthritis (Additional file 7). The expression of CXCR4 was significantly increased in CD62L^low ^population, indicating that CXCR4 expression was induced in activated T cells (Figure 4d). Furthermore, we examined the effect of anti-CD3 stimulation of LN cells on the CXCR4 expression in T cells. As shown in Figure 4e, CD44^high ^T-cell population contained a higher proportion of CXCR4^high ^cells, and the percentage of CXCR4^high ^cells in CD44^high ^T-cell population was higher in arthritic mice, consistent with the results in Figure 4d. These observations indicate that immune activation of T cells induces CXCR4 expression.

### T-cell accumulation in arthritic joints is dependent on CXCR4 expression in T cells

Next, we examined expression of CXCR4 in arthritic joints. Although CD3^+ ^T cells were found in both nonarthritic and arthritic joints, the number of T cells was significantly increased in arthritic joints, and most of the T cells expressed CXCR4 (Figures 5 and 6a). The expression of the *Cxcl12 *was also augmented in arthritic joints (Figure 6b). Furthermore, we found that the CXCR4^+ ^cell population in total T cells was much higher in the inflammatory sites of affected joints than in LNs (Figure 6c), indicating that CXCR4^+ ^cells accumulated in the inflammatory sites. It is remarkable that some CD3^+ ^T cells were closely apposed to SDF-1-positive cells in arthritic joints (Additional file 8), consistent with the idea that SDF-1 attracted these CXCR4-expressing cells.

To show whether T-cell accumulation in the joints during the development of CIA depends on the CXCR4/SDF-1 system, T cells from *Cxcr4^flox/flox^*/*Lck-Cre *mice and *Cxcr4^+/+^*/*Lck-Cre *mice were labeled with radioisotopes and injected into IIC/CFA-immunized mice, and T-cell migration into joints was analyzed (Figure 6d). Recovery of radioisotope-labeled T cells in affected joints was significantly decreased by the deficiency of CXCR4. These data clearly indicate that activated T cells were accumulated in inflamed joints in a CXCR4-dependent manner.

## Discussion

CIA is a model for autoimmune arthritis characterized by autoantibody production and massive infiltration of effector cells to joints [29,30]. However, immunization with native murine IIC shows a much lower incidence of arthritis in DBA/1J mice [31], and immunization with heterologous IIC, such as chicken or bovine, shows a much higher incidence in CIA. A peptide from chicken IIC (aa. 256 to 270) is known as a major immunodominant epitope [31,32], and the difference between heterologous IIC and murine IIC, which is important in the development of arthritis, is the presence of a glutamic acid at position 266 in chicken IIC. The T-cell response to this epitope is suggested to break tolerance to induce responses toward homologous epitopes in murine IIC. Bovine IIC (256-270)-specific T cells also crossreact with mouse IIC [32]. Interestingly, human IIC also shares this immunodominant epitope, because chicken IIC-reactive T-cell hybridoma responds to human IIC [33]. Furthermore, anti-human IIC antibodies purified from RA patients' sera react with chick and bovine IIC [34]. Thus, it is believed that chicken IIC-induced CIA is a good model for human RA.

It was reported that the proportion of CXCR4-expressing memory T cells and the expression of the ligand, SDF-1, are increased in RA synovium, suggesting that the SDF-1-CXCR4 system is involved in the pathogenesis of RA [11,12,35]. However, the precise functional roles of this molecule in T cells remains to be elucidated. In this study, we showed that the incidence, but not the severity, of CIA was significantly decreased in *Cxcr4^flox/flox^*/*Lck-Cre *mice, indicating that CXCR4 expression in T cells is important for the development of CIA. CXCR4 expression was enhanced in activated/memory T cells, and CXCR4-deficient T cells were defective in SDF-1-induced chemotaxis. Both CXCR4 expression in T cells and SDF-1 expression in the affected joints was augmented during the development of CIA, and CXCR4-expressing T cells were accumulated in the inflammatory sites where SDF-1 was strongly expressed. Importantly, on induction of CIA, the proportion of CXCR4-expressing T cells was increased in the inflammatory sites compared with that in the draining LNs, and infiltrated T cells were closely apposed with SDF-1-expressing cells. Although immunization with CFA without IIC enhanced CXCR4 expression in T cells, this could not induce arthritis, suggesting that CXCR4 expression in IIC-specific T cells is important for the induction of arthritis. Thus, the SDF-1-CXCR4 system in T cells is important to recruit memory T cells to the site of inflammation, rather than to the LNs. Because cytokines such as IL-15 and TGF-β can induce and/or sustain the expression of CXCR4 in T cells [12,36], cytokines from activated LN cells in IIC-immunized mice may enhance CXCR4 expression. Consistent with this notion, we showed that the antibody production and T-cell response against IIC were normal in *Cxcr4^flox/flox^*/*Lck-Cre *mice.

We suggest that CXCR4 expression in T cells plays an important role in mediating T-cell recruitment to inflamed sites. However, the importance of T cells, especially the recruitment of T cells to inflamed sites in the development of CIA, has been ignored for a long time. This is because adoptive transfer experiments suggest the importance of antibodies, but not T cells, in the development of arthritis [29,31]. CIA can be transferred by serum from arthritic mice, whereas T-cell transfer from arthritic mice to normal or lymphopenic mice cannot induce the development of arthritis [29,31]. However, it was reported that transfer of splenocytes from arthritic mice to SCID mice can induce arthritis [37]. In this experiment, the incidence of arthritis was greatly decreased when either T cells or B cells were depleted from the splenocytes. These observations suggest that T cells are involved in the development of arthritis, although T cells alone cannot induce arthritis. Furthermore, the involvement of T cells is reported in a collagen-antibody-induced arthritis (CAIA) model, in which arthritis is induced by the administration of collagen-specific antibodies [38]. This model has been considered to be independent of T cells, because T cell-deficient mice were susceptible to CAIA [39]. However, injection of collagen-specific T cells to CAIA-induced mice prolonged the disease [39], and T-cell suppression by CTLA-4 Ig treatment [40] or CTLA-4 Ig and cyclosporine treatment [41] in a later phase of CAIA suppressed the severity of arthritis, indicating that T cells play a role in CAIA as an amplifier. Accordingly, the following pathogenic processes are suggested. On production or administration of antibodies specific to IIC, these antibodies may activate mast cells by forming complexes with complements on cartilage IIC in joints, leading to the secretion of inflammatory cytokines and chemokines, including IL-1, similar to that reported in K/BxN arthritic mice [42]. These inflammatory mediators may activate synovial cells to recruit effector T cells, which can secrete various inflammatory mediators on activation with endogenous IIC, resulting in the amplification of inflammation. Our results indicate that CXCR4-SDF-1 interaction plays an important role in recruiting effector T cells into inflamed sites.

Although it was reported that CXCR4 signaling enhances T-cell activation and proliferation in a synergistic manner with TCR signaling [10,27,28,43], the proliferative response of LN cells from IIC-immunized *Cxcr4^flox/flox^*/*Lck-Cre *mice was normal on stimulation with IIC, and the cytokine production from these cells was also normal. We also did not detect any effects of SDF-1 on the proliferative response of IIC-immunized T cells to IIC stimulation. This apparent discrepancy might be caused by the difference of the experimental conditions; anti-CD3 antibodies were used to stimulate T cells in the previous reports [10,27,28,43], whereas whole LN cells, including antigen-presenting cells, were activated with natural antigens in our experiments. Weak costimulatory activity of CXCR4 may be masked by the strong costimulatory signaling from the antigen-presenting cells.

Recently, it was reported that CXCR4 is involved in the β-selection during thymic T-cell development in T cell-specific *Cxcr4*-deficient mice [44]. In contrast to this report, no significant abnormality was detected in the T-cell population and cell number in lymph nodes and the spleen between *Cxcr4^flox/flox^*/*Lck-Cre *mice and *Cxcr4^+/+^*/*Lck-Cre *mice (Additional file 2d and 2e), although in the thymus, the T-cell number was decreased without affecting the CD4^+ ^or CD8^+ ^cell content in *Cxcr4^flox/flox^*/*Lck-Cre *mice. We think this difference from the previous report was caused by the difference of the *Lck-Cre *transgenic mouse line we used. We found that, in our *Cxcr4^flox/flox^*/*Lck-Cre *mouse thymus, CXCR4 expression still remained in the DN3 stage (Additional file 2c), suggesting that this remaining CXCR4 rescued potential defects in the thymic β-selection.

*Cxcr4*-deficient mice developed arthritis with very low incidence (two of 11), but the severity of arthritis was comparable to that of control mice (Figure 1b-d). These data suggest that CXCR4 is required for the initiation of inflammation by recruiting IIC-specific T cells into inflammatory sites. However, IIC-specific effector T cells would migrate into joints by the action of other chemokines or even by chance, without involvement of CXCR4, although the frequency may be low. Then these T cells are activated with IIC in the joints and produce various cytokines and chemokines, which recruit or activate other inflammatory cells, such as neutrophils. Therefore, once inflammation starts, other chemokines and cytokines may play more important roles than CXCR4, or other chemokines may substitute for the role of CXCR4 in the later stages of inflammation, resulting in the development of arthritis in *Cxcr4^flox/flox^*/*Lck-Cre *mice. In support for this contention, we showed that most of the inflammatory cells infiltrated in inflammatory sites were neutrophils, and only a part of them were CD3^+ ^T cells (Figure 5). Furthermore, we showed that CCR6, which is important for the development of arthritis in SKG mice by enhancing the migration of Th17 cells, was expressed normally in *Cxcr4*-deficient T cells (Figure 3e). The expression of CD62L, which is important for T-cell migration to peripheral tissues, was also normal (Figure 3e).

On induction of CIA, the expression of SDF-1 was augmented. Regarding SDF-1, it was reported that inflammatory cytokines, such as IL-17 [45] and IL-18 [46], can induce production of this molecule by fibroblast-like synoviocytes and stromal cells in the RA synovium. Conversely, CXCR4 is induced by TGF-β and IL-15 [36]. Thus, the cytokine production associated with the immune response against IIC may activate the expression of these molecules, resulting in trapping of activated T cells and recruitment of new T cells to the inflammatory sites. Because SDF-1-CXCR4 system is also active in B cells to activate and to migrate [47], in osteoclasts to produce MMP-9 [48], in chondrocytes to produce MMP-3 [49], in endothelium to induce angiogenesis [50], and in Th1 cells to migrate into synovial tissues [35], this system may play very important roles in a multiple ways in the development of RA. Therefore, these findings highlight the SDF-1-CXCR4 system as a good target for the treatment of RA.

## Conclusions

We showed that CXCR4 expressed in T cells plays an important role in the development of CIA by recruiting activated/memory T cells into the inflammatory sites.

## Abbreviations

IIC: type II collagen; CIA: collagen-induced arthritis; KO: knockout; LN: lymph node; RA: rheumatoid arthritis.

## Competing interests

The authors declare that they have no competing interests.

## Authors' contributions

SC mainly contributed throughout this work in collaboration with KS, NF, and SS. Immunohistochemical analysis was operated in collaboration with KBK and AI. BC generated *Cxcr4^flox/flox ^*/*Lck-Cre *mice. YI organized and supervised the project and edited the draft manuscript. All authors read and approved the final manuscript.

## Supplementary Material

Additional file 1 **Supplemental Methods**. Materials and methods that were used in supplemental figures are described in Additional file 1. Click here for file

Additional file 2 **Generation of T cell-specific CXCR4-deficient mice**. **(a) ***Cxcr4 *gene in the thymus. *Cxcr4^flox/flox^/Lck-Cre, Cxcr4^flox/flox^*, and wild-type DBA/1J mice was analyzed by Southern blot analysis, as described in Supplemental Methods in Additional File 1. In brief, genomic DNA from thymocytes was digested with *Sca*I/*Xba*I, and hybridized with 0.8 kb *Sca*I fragment as a 5' probe at 42°C for overnight. Deleted allele, 6.1 kb (*Cxcr4^flox/flox^/Lck-Cre*); knockin allele, 8.3 kb (*Cxcr4^flox/flox^*); and wild-type allele, 10.1 kb (WT). **(b) **CXCR4 expression in thymocytes was assessed with FACS. **(c) **Thymocytes were stained with antibodies against CD4, CD8, CD25, CD44, and CXCR4, and DN3 (CD25^+^CD44^- ^CD4^-^CD8^-^), DN4 (CD25^-^CD44^- ^CD4^-^CD8^-^), DP (CD4^+^CD8^+^), and DN (CD4^-^CD8^-^) cells were gated. CXCR4 expression was detected in DN3 and DN4 stages, although the intensity was somewhat decreased, and only a little CXCR4 expression was detected at the DP stage. Representative data from five *Cxcr4^+/+^/Lck-Cre *mice and five *Cxcr4^flox/flox^/Lck-Cre *mice are shown. **(d) **T-cell numbers (left column) and populations (right column) in the thymus, LNs, and spleen were determined with flow cytometry. Means and SDs of data from four *Cxcr4^+/+^/Lck-Cre *mice (white bar) and three *Cxcr4^flox/flox^/Lck-Cre *mice (black bar) are shown. *P < 0.05, with Student's *t *test. **(e) **The proportion of CD3^+ ^T cells in LNs in normal mice and IIC-immunized mice (day 7) were analyzed with flow cytometry. Means and SDs of data from three *Cxcr4^+/+^/Lck-Cre *mice (white bar) and three *Cxcr4^flox/flox^/Lck-Cre *mice (black bar), which were immunized 7 days before, are presented. Data are representative of more than two independent experiments. Click here for file

Additional file 3 **B-cell response is normal in *Cxcr4^flox/flox^/Lck-Cre *mice**. **(a) **IIC-specific IgG antibody titer in sera was measured 7 days after IIC immunization with ELISA. Each circle represents one mouse. **(b) **Proliferative response of B cells was measured after stimulation with IgM. LN cells from IIC-immunized mice (four *Cxcr4^+/+^/Lck-Cre *mice and four *Cxcr4^flox/flox^/Lck-Cre *mice) were stimulated with/without 1 μg/ml of anti-IgM antibody for 3 days, and proliferative response was measured with [^3^H]-thymidine incorporation for 6 hours. Click here for file

Additional file 4 **T cells from draining LNs are normally activated in *Cxcr4^flox/flox^/Lck-Cre *mice after IIC immunization**. LN cells from IIC/CFA-immunized mice were stimulated with or without 100 μg/ml of IIC at 37°C for 72 hours, and the expression of CD25, CD62L, and CCR6 on CD4^+ ^cells was analyzed with FACS. Representative data from three independent experiments are shown, and all the data are summarized in Figure 3e. Click here for file

Additional file 5 **T-cell recall response against IIC is not enhanced by SDF-1**. One week after intradermal immunization with chicken IIC/CFA, LN cells collected from three DBA/1J mice were cultured in fresh medium for 3 hours and stimulated with SDF-1 (100 ng/ml) for 2.5 hours. Then these cells were cultured in the presence or absence of 50 μg/ml of denatured chicken IIC for 72 hours. Proliferative response was measured with [^3^H]-thymidine incorporation in triplicates, and averages and SDs of triplicates are shown. Data are representative of two independent experiments. Click here for file

Additional file 6 **CXCR4 expression is elevated in LN T cells from arthritic mice**. CXCR4 expression in draining LN CD4^+ ^T cells from CIA-induced mice was examined with flow cytometry. **(a) **CXCR4 expression in CIA-induced arthritic DBA/1J mice (arthritic), or nontreated DBA/1J (normal) mice. Representative data among five normal mice and nine arthritic mice are shown. **(b) **Statistical analysis of CXCR4 expression. Each circle represents an individual mouse, and averages and SDs are shown. *P < 0.05, Student's *t *test. Click here for file

Additional file 7 **Immunization with CFA without IIC cannot induce CIA**. DBA/1J mice were immunized with IIC and CFA (*n *= 7), or CFA only (*n *= 4). **(a) **Incidence of CIA. **(b) **T-cell proliferative response against IIC stimulation. Draining LN cells were stimulated with IIC for 72 hours, and the proliferative response was measured with [^3^H]-thymidine incorporation for 6 hours. Representative data from two similar experiments are shown. Click here for file

Additional file 8 **T cells are closely apposed to SDF-1-expressing cells in arthritic joints**. The expression of SDF-1 and CD3 was assessed in arthritic joints with immunohistochemistry. Green, SDF-1; red, CD3; and blue, nuclei. Representative data from two arthritic mice are shown. Click here for file
