# Supplementary material for: CXC chemokine receptor 4 expressed in T cells plays an important role in the development of collagen-induced arthritis
Source: Arthritis Res Ther. 2010 Oct 12;12(5):R188. doi: 10.1186/ar3158 (PMC2991023; doi:10.1186/ar3158)
Supplement: Additional file 1 — Supplemental Methods. Materials and methods that were used in supplemental figures are described in Additional file 1. [file ar3158-S1.doc]

**Supplemental Methods**

**Southern blot analysis**

To confirm the deletion of *Cxcr4* gene, southern blot analysis was performed as described previously [51]. Briefly, genomic DNA from the thymus was digested with Sca I and Xba I, and separated by electrophoresis in a 0.9% agarose gel, and transferred to a GeneScreen Plus membrane (NEN, Boston, MA). Hybridization was carried out with ^32^P-labelled DNA probes made by Multiprimed DNA labeling system (Amersham Pharmacia Biotech, Buckinghamshire, England) for overnight at 42^o^C. The 0.8 kb Sca I fragment (indicated as 5’ probe in Figure 1A) was used as a probe. Wild-type allele: 10.1 kb, knock-in allele: 8.3 kb, and deleted allele: 6.1 kb.

**Analysis on B cell response in immunized mice**

B cell response in IIC-immunized mice was measured as follows. Inguinal and infra-axillary LNs were harvested from mice which were immunized with IIC and CFA for 7 days. Single cell suspension was prepared, and 3 x 10^5^ cells/well were cultured in the absence or presence of 10 μg/ml anti-IgM antibody (Cappel, West.Chester, PA, USA) in 96-well flat bottom plates (Falcon) for 72 h, followed by incorporation of 25 μg/ml of [^3^H]-thymidine for 6 h.

**Immunohistochemistry**

Frozen sections obtained from CIA-affected limbs were fixed in PBS with 4% paraformaldehyde for 30 min at room temperature, and blocked with 2% bovine serum albumin (Sigma) in PBS with 0.05% Tween 20. Then, the sections were stained with anti-murine CD3 (Abcam, Ab16044) and biotinylated anti-SDF-1α (BAF310, R&D systems, Minneapolis, MN, USA), and signal from SDF-1α was detected by streptavidin FITC (BD pharmingen). Nikon A1Rsi-TiE confocal microscope was used to visualize the slides.
