# Supplementary figures and images for: CXC chemokine receptor 4 expressed in T cells plays an important role in the development of collagen-induced arthritis
Source: Arthritis Res Ther. 2010 Oct 12;12(5):R188. doi: 10.1186/ar3158 (PMC2991023; doi:10.1186/ar3158)

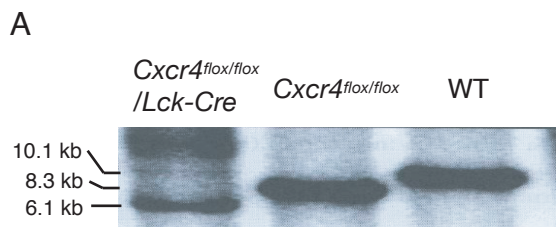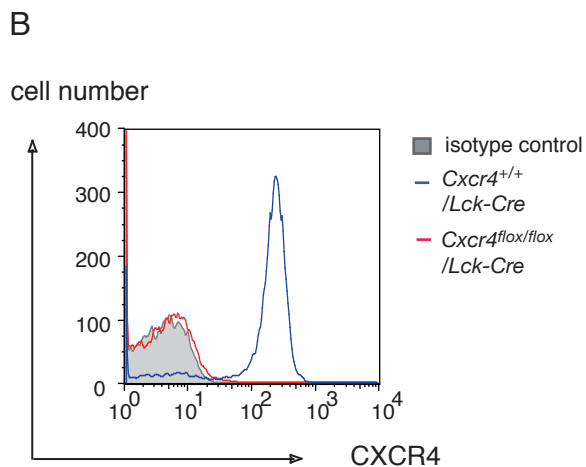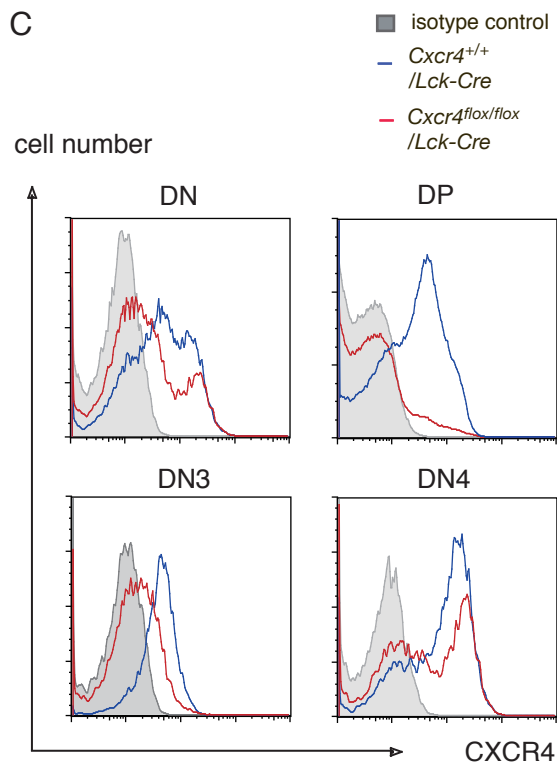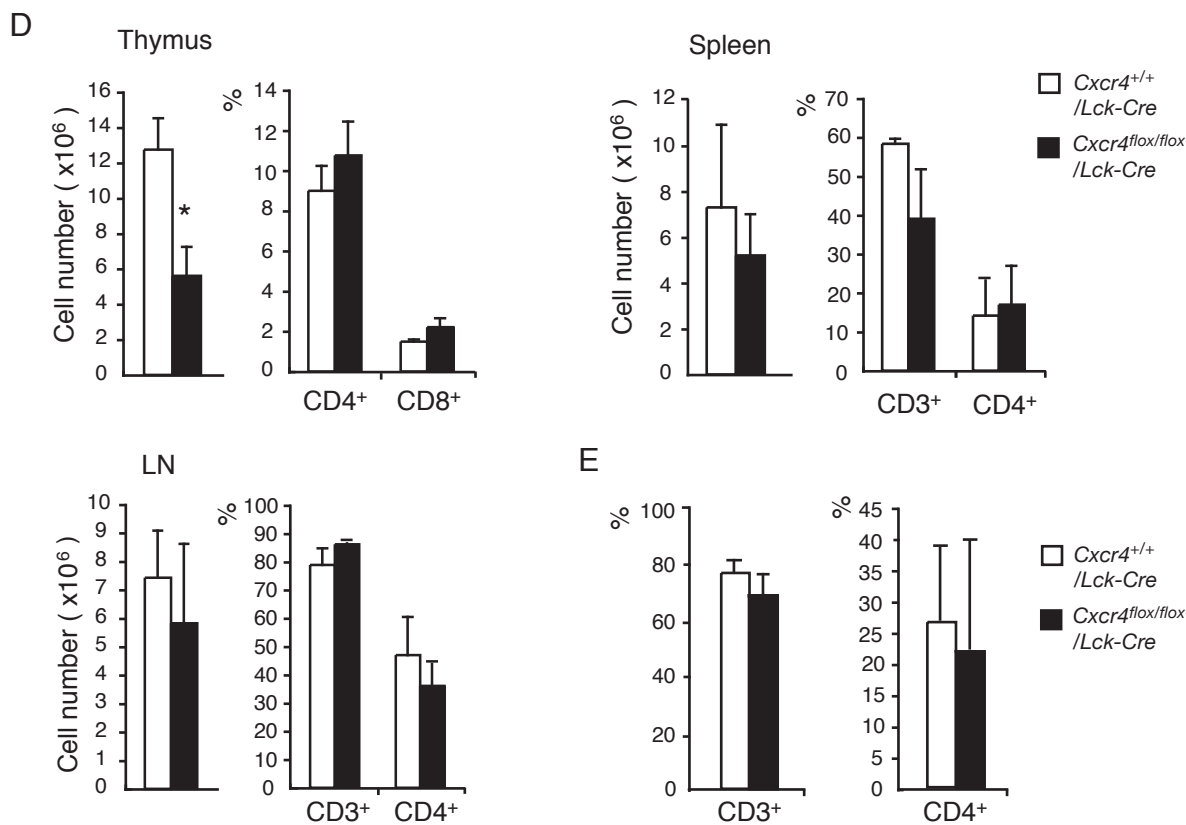

Supplement: Additional file 2 — Generation of T cell-specific CXCR4-deficient mice. (a) Cxcr4 gene in the thymus. Cxcr4flox/flox/Lck-Cre, Cxcr4flox/flox, and wild-type DBA/1J mice was analyzed by Southern blot analysis, as described in Supplemental Methods in Additional File 1. In brief, genomic DNA from thymocytes was digested with ScaI/XbaI, and hybridized with 0.8 kb ScaI fragment as a 5' probe at 42°C for overnight. Deleted allele, 6.1 kb (Cxcr4flox/flox/Lck-Cre); knockin allele, 8.3 kb (Cxcr4flox/flox); and wild-type allele, 10.1 kb (WT). (b) CXCR4 expression in thymocytes was assessed with FACS. (c) Thymocytes were stained with antibodies against CD4, CD8, CD25, CD44, and CXCR4, and DN3 (CD25+CD44- CD4-CD8-), DN4 (CD25-CD44- CD4-CD8-), DP (CD4+CD8+), and DN (CD4-CD8-) cells were gated. CXCR4 expression was detected in DN3 and DN4 stages, although the intensity was somewhat decreased, and only a little CXCR4 expression was detected at the DP stage. Representative data from five Cxcr4+/+/Lck-Cre mice and five Cxcr4flox/flox/Lck-Cre mice are shown. (d) T-cell numbers (left column) and populations (right column) in the thymus, LNs, and spleen were determined with flow cytometry. Means and SDs of data from four Cxcr4+/+/Lck-Cre mice (white bar) and three Cxcr4flox/flox/Lck-Cre mice (black bar) are shown. *P < 0.05, with Student's t test. (e) The proportion of CD3+ T cells in LNs in normal mice and IIC-immunized mice (day 7) were analyzed with flow cytometry. Means and SDs of data from three Cxcr4+/+/Lck-Cre mice (white bar) and three Cxcr4flox/flox/Lck-Cre mice (black bar), which were immunized 7 days before, are presented. Data are representative of more than two independent experiments. [file ar3158-S2.pdf]

A

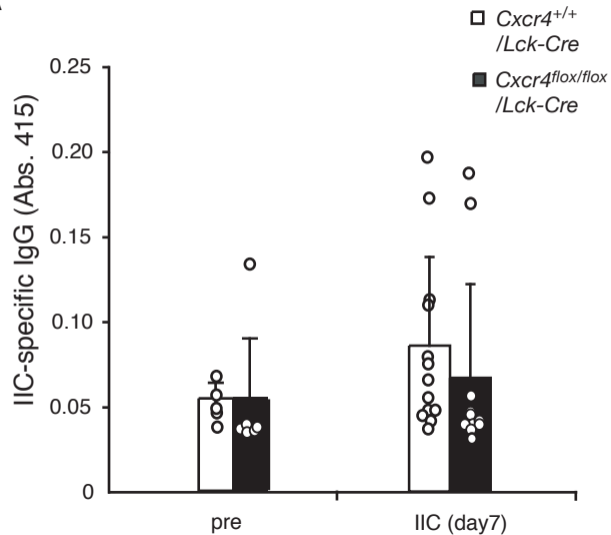

B

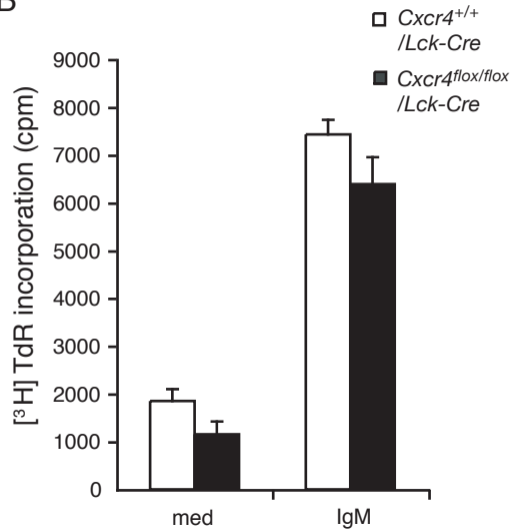

Supplement: Additional file 3 — B-cell response is normal in Cxcr4flox/flox/Lck-Cre mice. (a) IIC-specific IgG antibody titer in sera was measured 7 days after IIC immunization with ELISA. Each circle represents one mouse. (b) Proliferative response of B cells was measured after stimulation with IgM. LN cells from IIC-immunized mice (four Cxcr4+/+/Lck-Cre mice and four Cxcr4flox/flox/Lck-Cre mice) were stimulated with/without 1 μg/ml of anti-IgM antibody for 3 days, and proliferative response was measured with [3H]-thymidine incorporation for 6 hours. [file ar3158-S3.pdf]

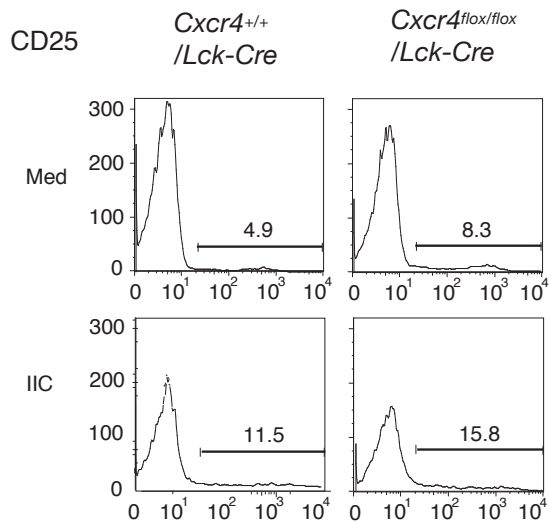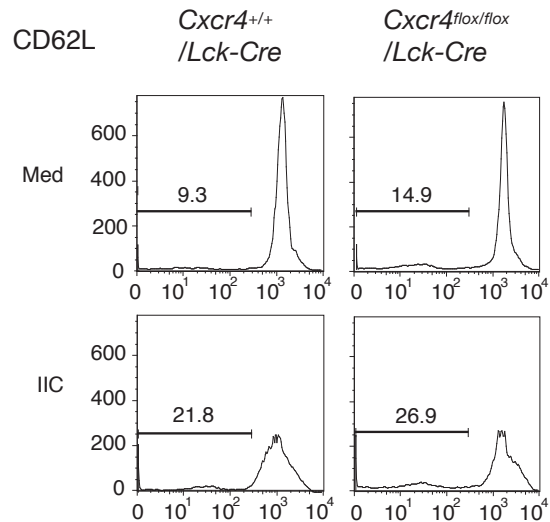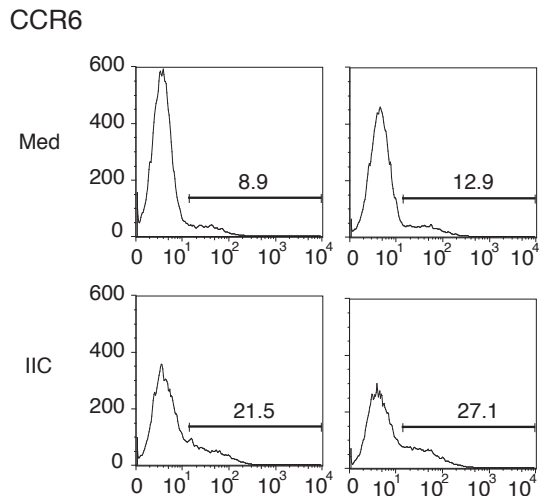

Supplement: Additional file 4 — T cells from draining LNs are normally activated in Cxcr4flox/flox/Lck-Cre mice after IIC immunization. LN cells from IIC/CFA-immunized mice were stimulated with or without 100 μg/ml of IIC at 37°C for 72 hours, and the expression of CD25, CD62L, and CCR6 on CD4+ cells was analyzed with FACS. Representative data from three independent experiments are shown, and all the data are summarized in Figure 3e. [file ar3158-S4.pdf]

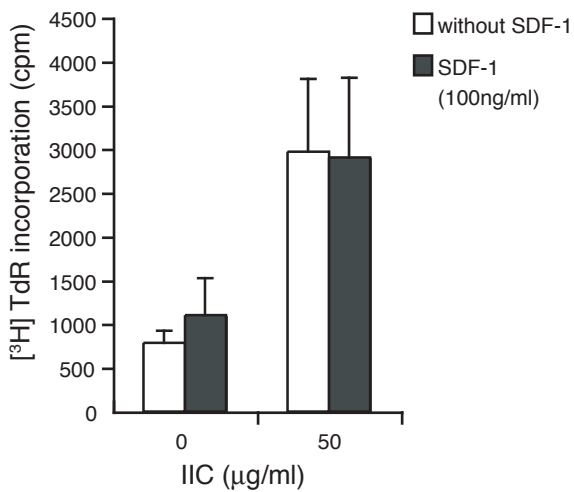

Supplement: Additional file 5 — T-cell recall response against IIC is not enhanced by SDF-1. One week after intradermal immunization with chicken IIC/CFA, LN cells collected from three DBA/1J mice were cultured in fresh medium for 3 hours and stimulated with SDF-1 (100 ng/ml) for 2.5 hours. Then these cells were cultured in the presence or absence of 50 μg/ml of denatured chicken IIC for 72 hours. Proliferative response was measured with [3H]-thymidine incorporation in triplicates, and averages and SDs of triplicates are shown. Data are representative of two independent experiments. [file ar3158-S5.pdf]

A

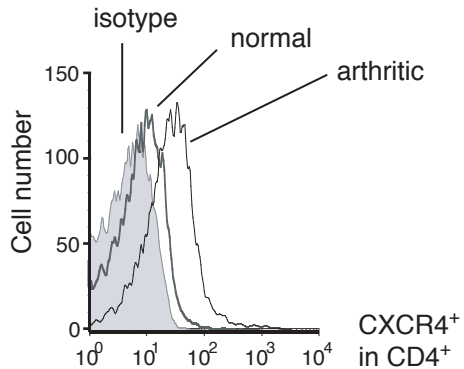

B

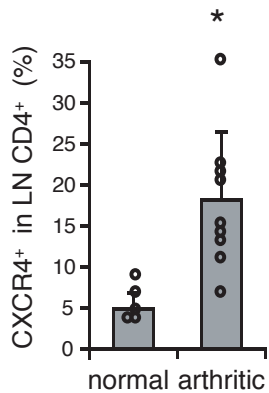

Supplement: Additional file 6 — CXCR4 expression is elevated in LN T cells from arthritic mice. CXCR4 expression in draining LN CD4+ T cells from CIA-induced mice was examined with flow cytometry. (a) CXCR4 expression in CIA-induced arthritic DBA/1J mice (arthritic), or nontreated DBA/1J (normal) mice. Representative data among five normal mice and nine arthritic mice are shown. (b) Statistical analysis of CXCR4 expression. Each circle represents an individual mouse, and averages and SDs are shown. *P < 0.05, Student's t test. [file ar3158-S6.pdf]

A

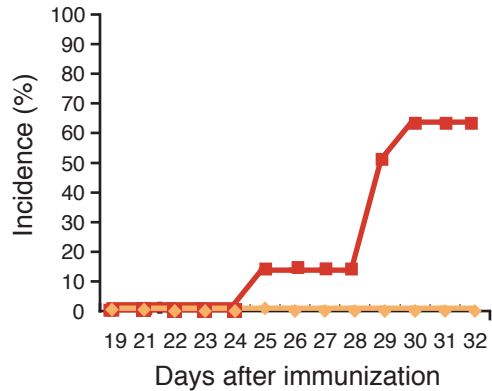

B

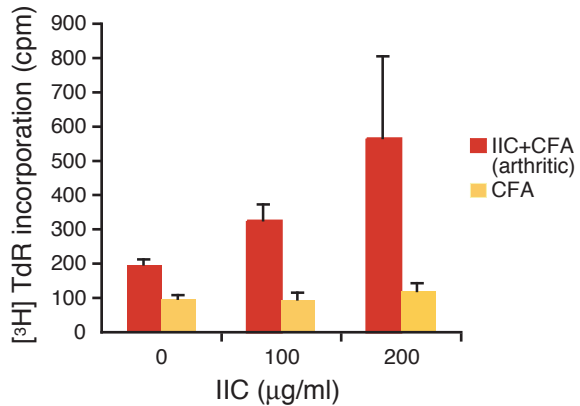

Supplement: Additional file 7 — Immunization with CFA without IIC cannot induce CIA. DBA/1J mice were immunized with IIC and CFA (n = 7), or CFA only (n = 4). (a) Incidence of CIA. (b) T-cell proliferative response against IIC stimulation. Draining LN cells were stimulated with IIC for 72 hours, and the proliferative response was measured with [3H]-thymidine incorporation for 6 hours. Representative data from two similar experiments are shown. [file ar3158-S7.pdf]
